# Structure-Based Cyclic Glycoprotein Ibα-Derived Peptides Interfering with von Willebrand Factor-Binding, Affecting Platelet Aggregation under Shear

**DOI:** 10.3390/ijms23042046

**Published:** 2022-02-12

**Authors:** Johana Hrdinova, Delia I. Fernández, Bogac Ercig, Bibian M. E. Tullemans, Dennis P. L. Suylen, Stijn M. Agten, Kerstin Jurk, Tilman M. Hackeng, Karen Vanhoorelbeke, Jan Voorberg, Chris P. M. Reutelingsperger, Kanin Wichapong, Johan W. M. Heemskerk, Gerry A. F. Nicolaes

**Affiliations:** 1 Department of Biochemistry, Cardiovascular Research Institute Maastricht (CARIM), Maastricht University, 6200 MD Maastricht, The Netherlands; rehakovajohana@gmail.com (J.H.); d.fernandezdelafuent@maastrichtuniversity.nl (D.I.F.); b.ercig@maastrichtuniversity.nl (B.E.); bibian.tullemans@maastrichtuniversity.nl (B.M.E.T.); d.suylen@maastrichtuniversity.nl (D.P.L.S.); s.agten@maastrichtuniversity.nl (S.M.A.); t.hackeng@maastrichtuniversity.nl (T.M.H.); c.reutelingsperger@maastrichtuniversity.nl (C.P.M.R.); k.wichapong@maastrichtuniversity.nl (K.W.); jwmheem722@outlook.com (J.W.M.H.); 2 Department of Molecular and Cellular Hemostasis, Sanquin-Academic Medical Center, 1011 LZ Amsterdam, The Netherlands; j.voorberg@sanquin.nl; 3 Platelet Proteomics Group, Center for Research in Molecule Medicine and Chronic Diseases (CiMUS), Universidad de Santiago de Compostela, 15782 Santiago de Compostela, Spain; 4 Center for Thrombosis and Hemostasis (CTH), University Medical Center, Johannes Gutenberg University Mainz, 55131 Mainz, Germany; Kerstin.Jurk@unimedizin-mainz.de; 5 Laboratory for Thrombosis Research, Interdisciplinary Research Facility Life Sciences, Katholieke Universiteit Leuven Campus Kulak Kortrijk, 8500 Kortrijk, Belgium; karen.vanhoorelbeke@kuleuven.be; 6 Synapse Research Institute, Kon. Emmaplein 7, 6214 AC Maastricht, The Netherlands

**Keywords:** glycoprotein Ib, in silico peptide design, platelets, thrombus, von Willebrand factor

## Abstract

The plasmatic von Willebrand factor (VWF) circulates in a compact form unable to bind platelets. Upon shear stress, the VWF A1 domain is exposed, allowing VWF-binding to platelet glycoprotein Ib-V-IX (GPIbα chain). For a better understanding of the role of this interaction in cardiovascular disease, molecules are needed to specifically interfere with the opened VWF A1 domain interaction with GPIbα. Therefore, we in silico designed and chemically synthetized stable cyclic peptides interfering with the platelet-binding of the VWF A1 domain per se or complexed with botrocetin. Selected peptides (26–34 amino acids) with the lowest-binding free energy were: the monocyclic mono- vOn Willebrand factoR-GPIbα InTerference (ORbIT) peptide and bicyclic bi-ORbIT peptide. Interference of the peptides in the binding of VWF to GPIb-V-IX interaction was retained by flow cytometry in comparison with the blocking of anti-VWF A1 domain antibody CLB-RAg35. In collagen and VWF-dependent whole-blood thrombus formation at a high shear rate, CLB-RAg35 suppressed stable platelet adhesion as well as the formation of multilayered thrombi. Both peptides phenotypically mimicked these changes, although they were less potent than CLB-RAg35. The second-round generation of an improved peptide, namely opt-mono-ORbIT (28 amino acids), showed an increased inhibitory activity under flow. Accordingly, our structure-based design of peptides resulted in physiologically effective peptide-based inhibitors, even for convoluted complexes such as GPIbα-VWF A1.

## 1. Introduction

The von Willebrand factor (VWF) plays an important role in the control of hemostasis and the onset of pathological arterial thrombosis [1,2]. Endothelial cells release VWF as ultra-large multimers into the blood, which are normally degraded by ADAMTS13 (a disintegrin and metalloprotease with thrombospondin type 1 motif 13) to a mixture of circulating large and small multimers [3,4]. In the blood, the VWF multimers assume a compact form, which prevents the binding to platelets [5]. However, at a high arterial shear rate and upon binding to collagen, the multimers elongate and undergo a conformational change. As a consequence, the VWF A1 domain becomes exposed and is able to bind to platelets via the large glycoprotein (GP)Ibα subunit of the GPIb-V-IX complex [6,7,8]. The antibiotic ristocetin induces a similar conformational change in VWF, which likewise induces GPIbα-VWF A1-binding [9,10]. The venom botrocetin binds to the VWF A1 domain, potentiating its interaction with the GPIbα chain [11].

Upon vascular damage or atherosclerotic plaque rupture, collagen-bound VWF, in a shear-dependent way, initiates thrombus formation through a fast onset of GPIb-V-IX-binding and platelet adhesion [12,13,14]. The transient adhesion of platelets is considered to be stabilized by integrin αIIbβ3, binding to the RGD-sequence in the VWF C4 domain, which facilitates platelet activation via signaling of the collagen receptor, glycoprotein VI (GPVI), and the adhesive collagen receptor, integrin α2β1 [15,16,17,18]. Platelet activation leads to outside-in signaling via αIIbβ3 binding to VWF and fibrinogen, which then facilitates stable platelet arrest, ensuing platelet aggregate formation. At shear rates of 1000 s^−1^ and higher, initial platelet adhesion is known to be triggered by the interaction between unrolled filamentous VWF A1 and GPIbα [19]. In addition, it is proposed that under flow, also homotypic VWF interactions support shear-dependent platelet aggregation and thrombus formation, i.e., at sites distant from the exposed collagen [15]. How these proposed multiple roles of VWF A1 contribute to the thrombosis process is still unclear.

For studying the molecular function of platelet GPIb-V-IX, it is important to have suitable drugs that interfere with the VWF-GPIbα interaction. Such drugs will also be of therapeutic interest; for example, for the treatment of patients with immune-mediated thrombotic thrombocytopenic purpura (iTTP). Such patients are known to have hyperactive ultra-large multimers of VWF due autoantibody-mediated inactivation of the clearance of the VWF-cleaving protease ADAMTS13 [20,21]. Candidate drugs tested in this respect are mostly targeting the VWF A1 domain and aim to suppress ultra-large VWF-dependent platelet activation. These include nano/antibodies such as caplacizumab and KB-VWF-006bi [22,23], and the aptamers ARC1779 [24], ARC15105 [25], and BT200 [26]. In addition, a snake venom-derived peptide antagonist, anfibatide (agkisacucetin), was shown to target the GPIb-V-IX complex [27]. Yet, the precise molecular effects of these drugs are incompletely understood and well-designed peptide-based antagonists affecting the VWF A1 domain may help to both improve the understanding of the multifaceted ligand interactions of platelets in arterial thrombus formation and, ultimately, provide a guide for novel therapeutics.

In recent years, peptide-based drugs are becoming of high interest because of their small size, ease of synthesis and modification, and good biocompatibility [28,29]. Peptides offer several advantages over antibody treatment as they provide limited steric hindrance and low manufacturing costs. In the present study, we designed and synthetized several peptides interfering with the ‘active’ conformation of VWF for functional studies of thrombus formation. The peptides were designed by utilization of in silico approaches, which we have successfully used to develop novel inhibitors for coagulation factor VIII [30], the signaling protein TRAF6 [31,32], the chemokine CCL5 [33], and extracellular histones [34,35]. For the present purpose, we employed two different strategies to design cyclic peptides that mimic the GPIbα-binding surface at the VWF A1 domain (ORbIT: vOn Willebrand factoR-GPIbα InTerference): one strategy used GPIbα as a structural template (mono-ORbIT) and the other used GPIbα in complex with botrocetin (bi-ORbIT). The latter mode is of interest since botrocetin reduces the VWF A1-GPIb off-rate by two-folds. It initially binds to VWF A1, after which the complexed botrocetin forms a new interaction with GPIbα [11]. Two best-performing binding peptides in silico were selected for chemical synthesis and subsequently experimentally tested for studying the GPIbα-VWF A1 interaction in vitro in a physiologically relevant whole-blood assay that operates at high wall-shear rate conditions. Based on the functional performance of the peptides, we performed another round of in silico design and synthesis using mono-ORbIT as a template and again evaluated the inhibitory potential of the new opt*-*mono-ORbIT towards the VWF A1-GPIbα axis.

## 2. Results

### 2.1. Design and Synthesis of Cyclic GPIbα-Mimicking Peptides Binding to the VWF A1 Domain

**Figure 1:**
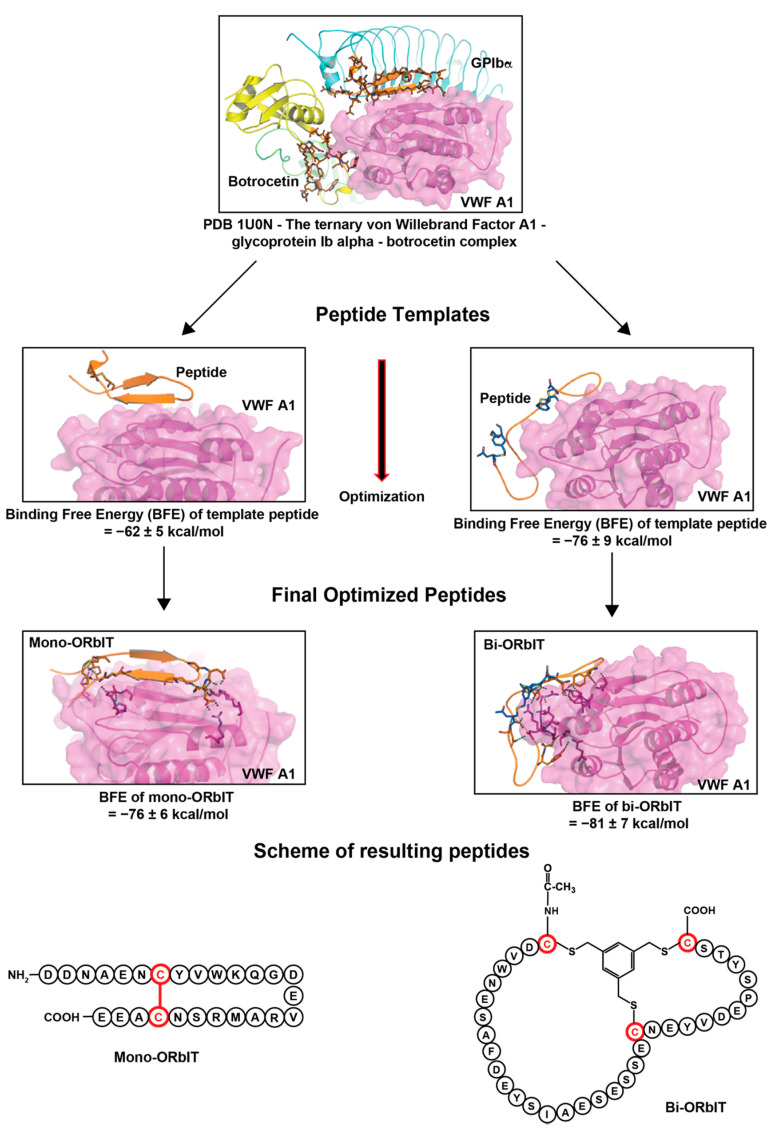
Design of GPIbα-mimicking peptides to interfere with VWF A1 domain. Molecular structures of the ternary complexes of GPIbα-VWF A1 and GPIbα-VWF A1–botrocetin (PDB code: 1U0N) were subjected to molecular dynamics simulations. Low energy structures shown of GPIbα (blue), botrocetin (yellow), and VWF A1 domain (purple). The indicated residues of GPIbα with (out) botrocetin identified as interacting with VWF A1 were used for the in silico design of (>100) mono and bi-cyclic peptides. All designed peptides were subjected to molecular dynamics simulations and binding free-energy (BFE) calculations. Peptide mutants with lowest BFE were selected for optimization and synthesis: mono-ORbIT (−76 ± 6 kcal/mol) and bi-ORbIT (−81 ± 7 kcal/mol).

The binding of VWF to platelet GPIb-V-IX is characterized by fast on and off-rates. Based on published molecular structures of the GPIbα chain in interaction with the exposed VWF A1 domain [11,36], we employed an in silico approach to design peptides with: (i) a sufficiently high affinity to interfere with the rapid interaction and (ii) a cyclic form to enhance their stability. As a structural template, we used the X-ray structure of the known ternary complex between GPIbα, the VWF A1 domain, and botrocetin (PDB code: 1U0N) with the purpose of designing a series of interfering mono and bi-cyclic peptides. As shown in Figure 1, for the design of the mono-cyclic peptides, we used, as a template, the binary interaction of GPIbα with the VWF A1 domain, while for the design of bi-cyclic peptides, we used that of the ternary complex between GPIbα, the VWF A1 domain, and botrocetin.

To predict the binding efficacy, we subjected published structures of the bimolecular GPIbα-VWF A1 complex and the trimolecular GPIbα-VWF A1-botrocetin complex, and we used molecular dynamic simulations to reveal key interacting regions at the complexed inter-molecular interfaces. The simulations were based on established and published protocols using standard parameter settings [37].

The known structure of the amino acids 221–246 of GPIbα, whose sequence interacts with VWF A1, was used to design a first set of interacting mono-cyclic peptides which was connected via a disulfide bond. For the ternary complex, amino acids 225–231 and 237–241 of GPIbα as well as residues 2069–2077 and 2089–2098 of botrocetin were used and designed as being connected by employing the method of the chemical linkage of peptides onto scaffolds (CLIPS) [38]. Per procedure, more than fifty peptides were designed in silico and all products were subjected to molecular dynamics simulation (5 ns) and binding free-energy (BFE) calculation to predict the relative binding affinity with the VWF A1 domain. The selected mono-cyclic peptide mono-ORbIT (26 amino acids) and the bi-cyclic peptide bi-ORbIT (34 amino acids) were found to exhibit the lowest BFE of **−**76 and **−**81 kcal/mol, respectively, thus predicting a high binding strength (Figure 1). These two peptides were synthetized and characterized (Table 1). The BFE value of the monocyclic GPIbα peptide was calculated as **−**35 ± 6 kcal/mol. The BFE of the botrocetin-based peptide sequence could not be calculated since it was composed of distinct small peptides derived from the botrocetin and the GPIbα chain.

For the Bi-ORbIT peptide, determination of sites for the formation with the linker occurred by a combination of structural analysis and per-residue energy decomposition analysis. Therein, we selected those amino acid residues that had a lesser contribution for interacting with the VWF A1 domain for mutation to conjugate with the linker. In this case, we rationally changed the positions of amino acid residues for connection by CLIPS. For the mono-ORbIT peptide, we did not optimize the disulfide bond positions.

Chemical synthesis of the linear peptide chains was performed by manual BOC-mediated solid-phase peptide synthesis. After cleavage from the solid support, the linear peptides were purified and cyclized. The cyclization was achieved by oxidation of two free cysteines to form a disulfide bond (mono-ORbIT) or by allowing the reaction of three free cysteines in the linear peptide chain with 1,3,5-tris(bromomethyl)benzene (T3) to form a 2-CLIPS constrained peptide (bi-ORbIT; Figures S1 and S2). The purified peptides were finally dissolved into saline water.

### 2.2. GPIbα-Derived Inhibitory Peptides Interfere with Ristocetin and Botrocetin-Induced VWF-Binding

As a proof-of-principle that the new cyclic peptides interfered with the binding of VWF to platelets, we made use of the ability of ristocetin and botrocetin to ‘open up’ and activate multimeric VWF, enabling it to bind to platelet GPIbα. The monoclonal antibody (mAb) CLB-RAg35 directed against the VWF A1 domain, previously developed in the van Mourik lab [39], was used as a reference agent. For quantification of the binding of plasma-derived VWF to platelets in the presence of ristocetin or botrocetin, we employed a recently optimized flow cytometric assay [10]. Platelets in diluted plasma (containing autologous VWF) were therefore incubated with ristocetin or botrocetin and after fixation, VWF-binding to the platelets was quantified by flow cytometry using a FITC-labeled anti-VWF antibody.

**Figure 2:**
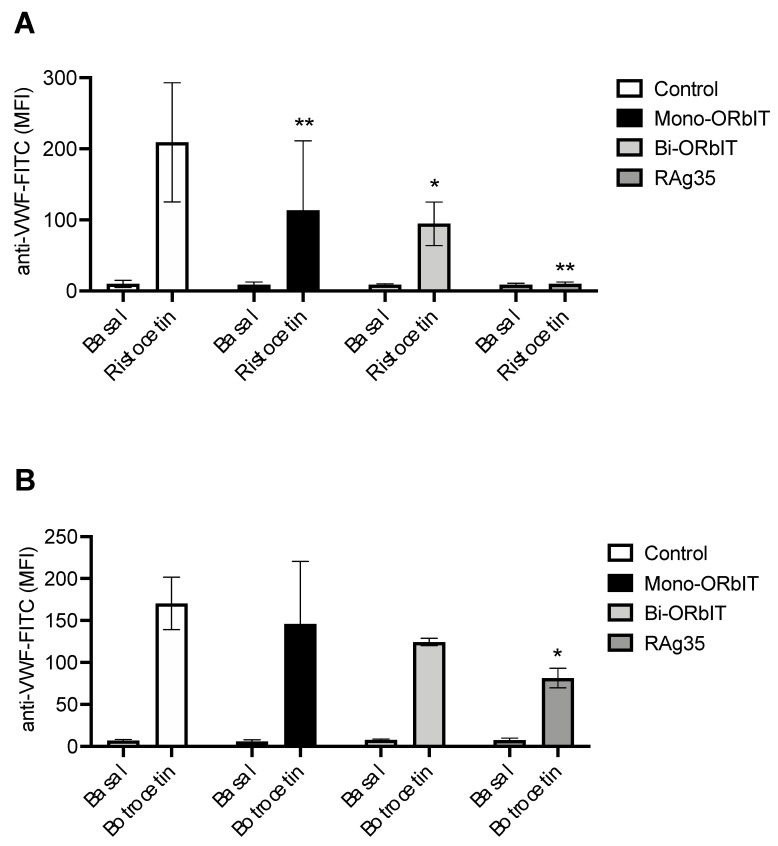
Reduced ristocetin and botrocetin-induced VWF-binding to platelet GPIb by mono-ORbIT and bi-ORbIT. Diluted platelet-rich plasma (PRP) containing tirofiban (1.25 µg/mL) was pre-incubated with the GPIbα-derived peptides (mono-ORbIT or bi-ORbIT at 200 µg/mL) or CLB-RAg35 mAb (RAg35, 5 µg/mL) for 10 min at room temperature. The mixtures were activated for 6 min with either ristocetin (0.5 mg/mL) or botrocetin (5 µg/mL). Platelet samples were fixed before labeling with fluorescein isothiocyanate (FITC)-conjugated anti-VWF Ab. For flow-cytometric analysis, a gating of selected single platelets was used and fluorescence was measured in the F1 channel. VWF-binding obtained as mean fluorescence intensities (MFI) for ristocetin (**A**) or botrocetin-stimulated platelets (**B**). Paired Student’s *t*-test, * *p* < 0.05, and ** *p* < 0.01 (n = three to five donors).

As indicated in Figure 2, we found that the binding of VWF-ristocetin to platelets was strongly inhibited by mono-ORbIT (49% fluorescence reduction vs. the control, *p* < 0.01) as well as by bi-ORbIT (58% fluorescence reduction vs. the control, *p* < 0.05). On the other hand, we observed only a minor reduction in the binding of VWF-botrocetin to platelets with mono-ORbIT (14% fluorescence reduction vs. the control) or bi-ORbIT (27% fluorescence reduction vs. the control). In comparison, the CLB-RAg35 mAb almost completely prevented the ristocetin-dependent VWF-binding (95% fluorescence reduction vs. the control, *p* < 0.01) and strongly inhibited the botrocetin-induced VWF-binding (52% inhibition vs. the control, *p* < 0.05).

### 2.3. Interference in the VWF A1 Domain Interaction with GPIbα, Affecting Thrombus Formation at High-Shear Flow

To better understand the role of the VWF-GPIbα interaction at high-shear arterial flow conditions, we applied a previously validated method of microspot whole-blood thrombus formation. This multiparameter test provides simultaneous information on the processes of flow-dependent platelet adhesion, aggregation, and activation [40,41]. For this study, we used a combination of three microspots, encompassing a wide range of GPIb-V-IX dependent surfaces, i.e., collagen-I (Horm-type collagen-I as a standard, microspot M1) [41], human collagen-III (with high VWF-binding, M2) [8], and the combination of VWF and fibrinogen (M3) [40]. At first, we tested the anti-A1 domain CLB-RAg35 antibody, which is described to inhibit VWF-dependent platelet adhesion to the sub-endothelial matrix [39,42]. The high-shear perfusion of whole-blood over microspots M1–3 resulted in different types of thrombi. Using multicolor microscopic imaging per microspot and standard image analysis, we phenotyped these thrombi according to eight different parameters at end-stage (Table 2). Brightfield images provided parameters informing on platelet adhesion (P1–2) and platelet aggregation or thrombus buildup (P3–5), while three-color fluorescence images (AF647 anti-CD62P mAb, P6; FITC anti-fibrinogen mAb, P7; and phosphatidylserine exposure AF568 annexin A5, P8) provided parameters of platelet activation.

**Figure 3:**
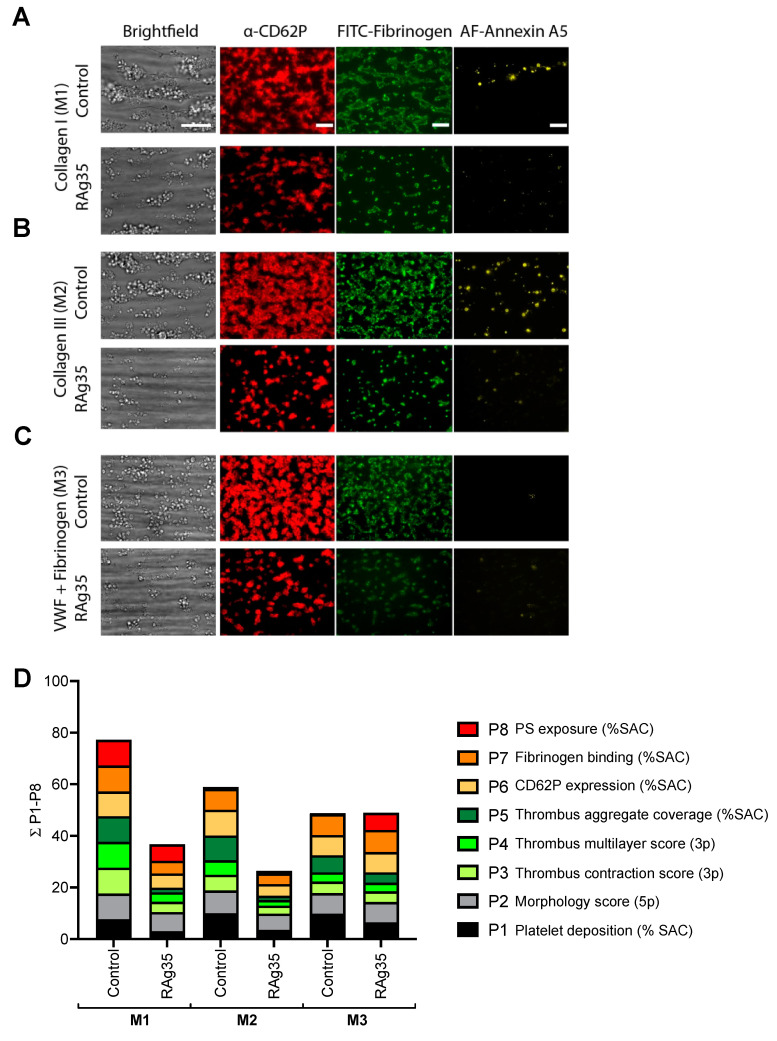
Thrombus and aggregation-reducing effect by blockage of the interaction of GPIbα with VWF A1 domain at high-shear flow. Whole-blood was preincubated for 10 min with CLB-RAg35 mAb (RAg35, 10 µg/mL) or equal volume of saline. Blood samples were flowed over microspots consisting of collagen-I (M1), collagen-III (M2), or VWF/fibrinogen (M3) for 3.5 min at wall-shear rate of 1600 s^−1^. (**A**–**C**) Representative brightfield microscopic images at end stage. Bars = 20 µm. Brightfield images were used for analysis of adhesion-related parameters, namely platelet deposition (P1) and morphological score (P2), and aggregation-related parameters (P3–5). End-stage three-color fluorescence images used for platelet activation assessment: CD62P expression (AF647 anti-CD62P mAb, P6), fibrinogen-binding (FITC anti-fibrinogen mAb, P7), and phosphatidylserine exposure (AF568 annexin A5, P8). (**D**) Cumulative representation of scaled values (0–10) per parameter. Color reflects adhesion parameters P1–2 (shades of black), aggregation parameters P3–5 (shades of green), and activation parameters P6–8 (shades of red). For details, see Figure S3.

Representative microscopic images from each of the three microspots under control conditions illustrated the marked differences in thrombus buildup (Figure 3A–C). This concerned the formation of larger-size platelet aggregates or thrombi on collagen-I (M1), smaller-size aggregates on collagen-III (M2), and mostly single platelets adhering to VWF/fibrinogen (M3). On all three surfaces, we detected noticeable platelet activation in terms of P-selectin expression (CD62P), fibrinogen-binding, and phosphatidylserine exposure, the latter of which, though, was highest on collagen-I (M1).

Quantification of the effects of the CLB-RAg35 mAb addition to blood, at the maximally effective dose of 5 µg/mL, indicated strong inhibitory effects on the formed thrombi (Figure 3D and raw data with statistics in Figure S3). More precisely, on collagen-I (M1), CLB-RAg35 intervention decreased the values of platelet deposition (P1, 40% of control, *p* < 0.0001) and the morphology score (P2, 73%, *p* < 0.0001). This was accompanied by a profound reduction in the platelet aggregation parameters, namely the thrombus contraction score (P3, 40%, *p* < 0.0001), thrombus multilayer score (P4, 36%, *p* < 0.0001), and platelet aggregate coverage (P5, 14%, *p* < 0.0001), and also by a reduction in the activation parameters of CD62P expression (P6, 56%, *p* < 0.0001) and fibrinogen-binding (P7, 52%, *p* < 0.001), while CLB-RAg35 did not have a significant effect on phosphatidylserine exposure (P8).

Essentially the same parameters were affected by CLB-RAg35 for the smaller-size thrombi formed on collagen-III (Figure 3D): platelet deposition (P1, 37% of control, *p* < 0.0001), morphology score (P2, 72%, *p* < 0.001), thrombus contraction score (P3, 58%, *p* < 0.01), thrombus multilayer score (P4, 44%, *p* < 0.001), platelet aggregate coverage (P5, 19%, *p* < 0.001), CD62P expression (P6, 45%, *p* < 0.001), and fibrinogen-binding (P7, 54%, *p* < 0.05). For the VWF/fibrinogen microspots (M3), causing mostly single-platelet adhesion (Figure 3D), only one parameter was significantly decreased by CLB-RAg35: platelet deposition (P1, 67% of control, *p* < 0.001). The phosphatidylserine exposure (P8) observed with CLB-RAg35 on the VWF/fibrinogen-coated surface (M3) points to a slightly increased platelet activation state on fibrinogen when the interaction with VWF is blocked. Jointly, these data revealed a major, unexpected role of GPIbα interaction with VWF A1 at a high shear rate, extending from VWF-dependent platelet adhesion to platelet aggregate and thrombus formation regardless of the type of collagen.

### 2.4. Designed GPIbα-Derived Peptides Interfering with Platelet Aggregation and Thrombus Formation under High-Shear Flow

**Figure 4:**
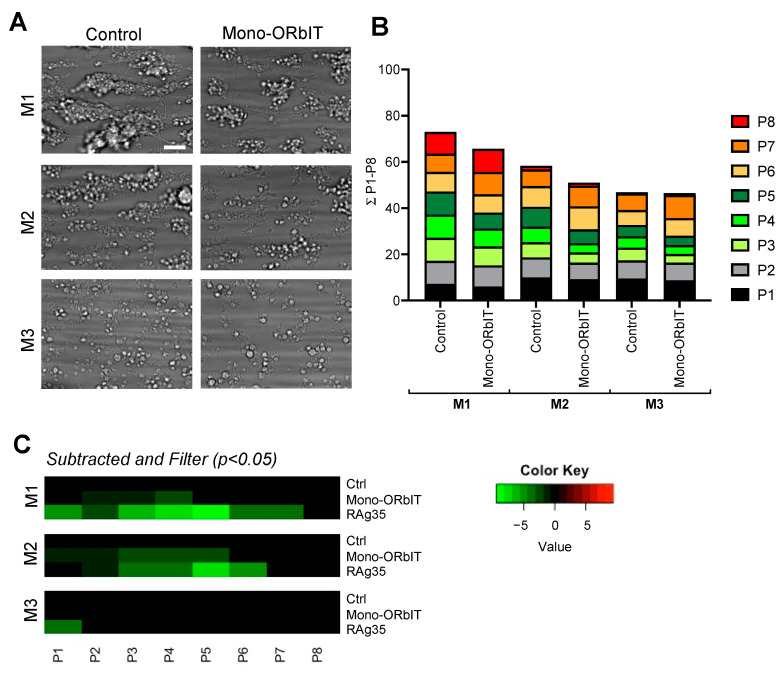
Suppressed thrombus formation and platelet aggregation by mono-ORbIT at high-shear flow. Whole-blood was preincubated for 10 min in parallel with mono-ORbIT (20 µg/mL), CLB-RAg35 mAb (RAg35, 10 µg/mL), or saline (control). Duplicate blood samples were flowed over microspots consisting of collagen-I (M1), collagen-III (M2), or VWF/fibrinogen (M3) for 3.5 min at wall-shear rate of 1600 s^−1^ and then stained, as seen in Figure 3. (**A**) Representative end-stage brightfield microscopy images per microspot in the absence or presence of mono-ORbIT. Bar = 10 µm. (**B**) Cumulative plots of scaled data per parameter (0–10), showing effect of mono-ORbIT. Platelet adhesion parameters: P1–2 (shades of black), aggregation parameters: P3–5 (shades of green), and activation parameters: P6–8 (shades of red). (**C**) Heatmap representation of control-subtracted data for mono-ORbIT and CLB-RAg35. Color coding indicates decrease (green) or increase (red) in comparison to control runs. Mean values were compared per each blood sample using a paired Student’s *t*-test: (n = seven donors). For raw data, see Table S1.

To assess the inhibitory potential of the newly designed mono-ORbIT (low concentration of 20 µg/mL) in whole-blood thrombus formation, we applied the same multiparameter output, as described above. Representative end-stage microscopic images indicated the presence of smaller-size thrombi and less-deposited platelets for M1–3 (Figure 4A). Overall, across blood from the seven donors tested, we noticed a consistent reduction with mono-ORbIT in the platelet aggregation for the two thrombus-forming microspots M1–2. Quantification indicated a reduction of the scaled parameters for platelet adhesion (P1–2) and aggregation (P3–5; Figure 4B). Representation of effect sizes as a subtraction heatmap showed a consistent inhibitory pattern for mono-ORbIT (Figure 4C). In detail, for collagen-I (M1), we observed a significant reduction of P2–4 to 77–92% (*p* < 0.05) of the control condition. For collagen-III (M2), the adhesion parameters (P1–2) were significantly reduced to 92% (*p* < 0.05) and 84% (*p* < 0.05), while the aggregation parameters (P3–5) were decreased to 60–69% (*p* < 0.05) of the control. For the VWF/fibrinogen microspot, where only single platelets adhered, none of the parameters were changed. Platelet activation markers (P6–8) were not significantly affected by this peptide.

Dose–response curves indicated a persistent inhibitory effect of mono-ORbIT at up to ten times higher concentration (Figure S4; raw data in Table S1). 

**Figure 5:**
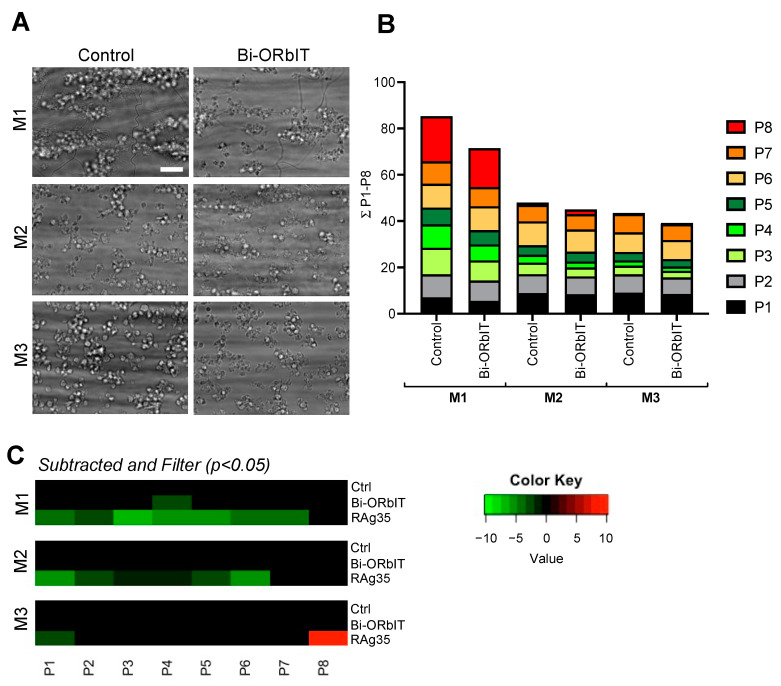
Moderately reduced platelet aggregation by bi-ORbIT at high-shear flow. Whole-blood was preincubated for 10 min in parallel with bi-ORbIT (20 µg/mL), CLB-RAg35 mAb (RAg35, 10 µg/mL), or saline (control). Experimental setup and analysis as shown in Figure 3. (**A**) Representative end-stage brightfield microscopy images per microspot in the absence or presence of bi-ORbIT. Bar = 10 µm. (**B**) Cumulative plots of scaled data per parameter (0–10), showing the effects of bi-ORbIT. (**C**) Heatmap representation of control-subtracted data for bi-ORbIT and RAg35. Color coding indicates decrease (green) or increase (red) in comparison to control runs (*n* = five donors, paired Student’s *t*-test, *p* < 0.05). For raw data, see Table S2.

Regarding the bi-ORbIT peptide (20 µg/mL), effects on whole-blood thrombus formation were similar in sign but smaller than those of mono-ORbIT (Figure 5A). The overall pattern of the scaled parameters with blood from five donors pointed to an apparent reduction of the aggregation-related parameters (P3–5) for microspots M1–2 (Figure 5B). However, significance was only reached for P4 (thrombus multilayer score) on M1 (69% of control, *p* < 0.05). As shown in a subtraction heatmap (Figure 5C), parallel runs carried out with CLB-RAg35 gave an expected reduction in most of the observed parameters. Further analyses of the effects of bi-ORbIT at higher concentrations were hindered by its poor solubility. Raw data per parameters of thrombus formation per microspot are shown in Table S2.

### 2.5. Additional Optimization of Mono-ORbIT Peptide to Opt-Mono-ORbIT

**Figure 6:**
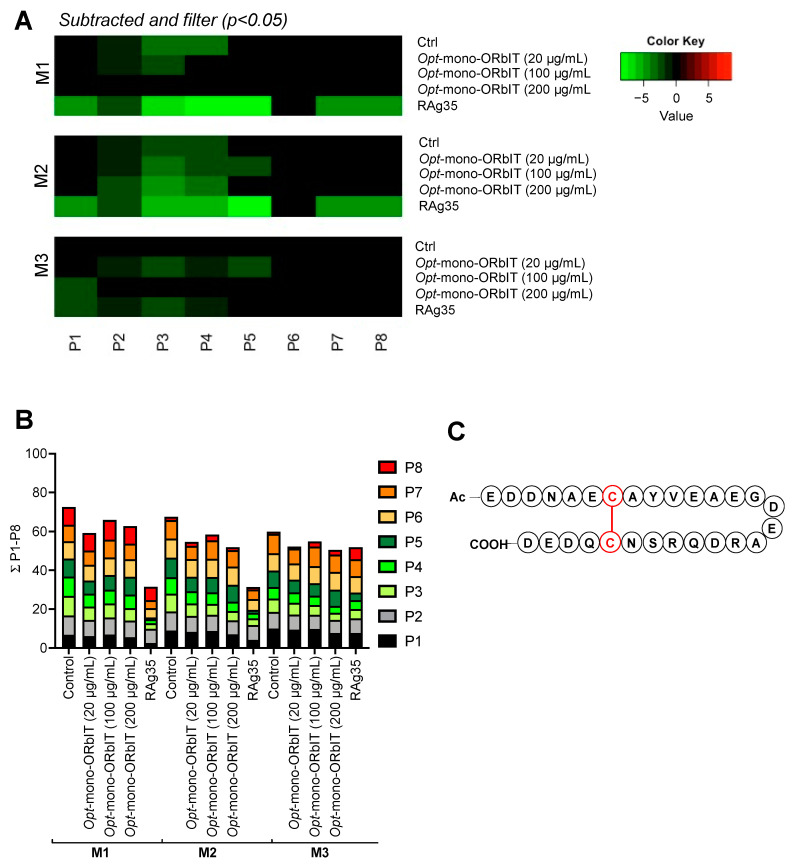
Additionally reduced platelet aggregation and thrombus formation by opt-mono-ORbIT at high-shear flow. Whole-blood was preincubated for 10 min in parallel with opt-mono-ORbIT (20, 100, and 200 µg/mL), CLB-RAg35 (RAg35, 10 µg/mL), or control (saline). Blood samples were flowed at 1600 s^−1^ and stained, as indicated in Figure 3. Mean values from individual blood samples were scaled 0–10 per parameter across all surfaces M1–3. (**A**) Heatmap representation of control-subtracted data. Color coding indicates decrease (green) or increase (red) in comparison to control runs. Filtering for relevant changes (n = three to seven donors, paired Student’s *t*-test, *p* < 0.05). (**B**) Cumulative plot of scaled data per parameter (0–10), showing effect of opt-mono-ORbIT. Platelet adhesion parameters: P1–2 (shades of black), aggregation parameters: P3–5 (shades of green), and activation parameters: P6–8 (shades of red). See further details in Table S3. (**C**) Schematic representation of opt*-*mono-ORbIT, in which a disulfide bond is indicated in red (C-C).

Given the twice as high inhibitory effects of RAg35 mAb on thrombus parameters, we reasoned that there is room for a higher efficacy of the synthetized peptides. We thus designed a set of novel mono-cyclic peptides in silico based on the mono-ORbIT peptide as a template. Similar protocols as described for mono-ORbIT and bi-ORbIT were applied. First, molecular dynamics simulations of several improved mono-cyclic peptides were performed for 5 ns of which the BFE was calculated. As some of the peptides gave comparable BFE values, further molecular dynamics simulations were performed for 100 ns to distinguish the theoretically most stable peptides for binding to the VWF A1 domain. Extended the analysis of these simulations and subsequent BFE calculations resulted in the selection of an improved peptide, namely opt-mono-ORbIT (Ac-EDDNAECAYVEAEGDEARDQR SNCQDED-COOH), which displayed the lowest BFE (−97.11 ± 8.44 kcal/mol) amidst a set of 20 mono-ORbIT-based peptides. The 28 amino-acid opt-mono-ORbIT was thus selected for synthesis. The peptide was cyclized with a disulfide bond between residues C_7_ and C_24_ (Figure 6C), and the opt*-*mono-ORbIT was spectrally analyzed for purity (Figure S5).

Flow cytometry indicated a notable inhibitory effect on VWF-platelet-binding in response to ristocetin (Figure S6). In the microfluidics system, blood treatment with opt*-*mono-ORbIT caused dose-dependently a stronger inhibition of thrombus formation than mono-ORbIT (Figure 6 and details in Table S3). On collagen-I (M1), consistent inhibition was observed with opt*-*mono-ORbIT (20 µg/mL) for the adhesion parameter P2 *(*83% of control, *p <* 0.05) and importantly for the aggregation parameters P3–4 (67–69%, *p* < 0.05). Regarding the collagen-III surface (M2), inhibition was also seen for platelet adhesion P2 (83%, *p* < 0.05) and platelet aggregation P3–4 (70–75%, *p* < 0.05). Increasing concentrations of opt*-*mono-ORbIT revealed a greater inhibitory potential in particular for spot M2 (Figure 6A,B). Inhibitory effects for the GPIbα specific surface M3 were only achieved at 100 µg/mL for P2–5 (77–85%, *p* < 0.05). Together, the results with the constructed mono-ORbIT and opt*-*mono-ORbIT point to consistent inhibitory effects on collagen/VWF-dependent platelet aggregation and thrombus formation, exceeding the effects on VWF-dependent platelet adhesion.

## 3. Discussion

### 3.1. Functional Efficacy of In Silico Designed GPIba Peptides Interfering in the Binding to the VWF A1 Domain

In order to explore a novel strategy for the development of the inhibitors of the interaction of VWF with platelet GPIb-V-IX, which is pathologically in iTTP, we designed novel peptide-based inhibitors to target the VWF A1 domain rather than to target the GPIb-V-IX complex on platelets. The advantage of this approach is that the binding site for GPIbα in the A1 domain is not accessible in circulating VWF multimers under physiological conditions. This approach hence may produce VWF inhibitors that are effective only after exposure of the A1 domain-binding site under shear stress or after interaction with vascular collagens, thereby theoretically leaving other VWF functions unchanged. For this purpose, we designed two distinct sets of peptides in silico, which resulted in the peptides mono-ORbIT (GPIbα-derived) and bi-ORbIT (botrocetin-GPIbα-derived). The rationale for the second design was that botrocetin not only binds VWF but also stabilizes the GPIbα-VWF A1 complex formation [9]. The two peptides, namely mono-ORbIT and bi-ORbIT, displayed, based on molecular dynamics simulations, a predicted low-binding free energy and hence were selected for chemical synthesis and functional evaluation.

When establishing their efficacy to interfere with the GPIbα-VWF A1 interaction by flow cytometry, mono-ORbIT indeed suppressed the binding of ristocetin and botrocetin-activated VWF to platelets by 49% and 14% fluorescence reduction, respectively. The synthesized bi-ORbIT displayed a stronger inhibitory effect on ristocetin-activated VWF (58% fluorescence reduction) than on botrocetin-activated VWF (27% fluorescence reduction). The reference CLB-RAg35 mAb inhibited ristocetin-induced VWF-binding to platelets by 95% and only partially inhibited the botrocetin-induced binding (52% fluorescence reduction). The generally lower inhibitory effect on botrocetin-induced binding observed for all inhibitors may be due to the mechanism of botrocetin action. Botrocetin first interacts only with VWF A1 and then slides around its A1 domain to form a new interface while interacting with GPIbα [11]. A higher peptide concentration than in the thrombus formation assay was used for the flow cytometric evaluation of the ristocetin and botrocetin- induced VWF-binding flow cytometry assay due to the absence of shear conditions which greatly enforce VWF-binding to GPIb-V-IX.

### 3.2. Role of VWF A1 Domain in Shear-Dependent Platelet Aggregation and Thrombus Formation on Collagen Surfaces

The two synthesized peptides were tested under physiologically relevant shear conditions in whole-blood using an established microfluidic-based platform [40]. With the established CLB-RAg35 mAb, we found that for the thrombogenic surfaces, collagen-I, and collagen-III, it suppressed not only the shear-dependent stable adhesion of platelets but also the parameters of platelet aggregate formation and thrombus size. In addition, we noted inhibitory effects of CLB-RAg35 on the secretion and fibrinogen-binding by the remaining platelets. The same pattern of changes in collagen-induced thrombus formation, although to a lesser extent, was also observed by the synthesized cyclic peptides, aimed to interfere in the VWF-GPIbα interactions. These results revealed an unexpected major role of VWF-binding to the platelet GPI-V-IX receptors in shear-dependent platelet activation and aggregation upon thrombus formation. An explanation for this finding is the earlier resolved formation of homotypic VWF-VWF interactions under shear conditions [15,39].

Both mono-ORbIT and bi-ORbIT appeared to reduce platelet aggregation and thrombus buildup under high-shear flow in a similar manner as CLB-RAg35. Yet, we noted a certain variation in the inhibitory effects among the blood samples tested, which may be due to inter-individual differences in VWF level and multimer composition, differences that are known to be related to blood type, genetic makeup, [43], or sex-related platelet responses [44,45]. In support of the current data, we and others have previously shown that the role of GPIbα in flow-dependent platelet aggregation was most prominent at high pathological wall-shear rate gradients, i.e., as present at stenotic sites in the arterial circulation [46,47]. A recently published single-chain antibody, scFV-A1, designed to target VWF activated by shear, was shown to play an inhibiting effect in stenotic chambers, i.e., at sites of sharp shear rate changes [48]. Therefore, stenotic flow chambers may be a useful tool to further study the effects of the present cyclic peptides under conditions of pathological VWF-dependent platelet activation.

Both mono and bi-ORbIT peptides displayed VWF A1-GPIbα inhibitory effects that were smaller in effect size than those obtained with the anti-VWF mAb. Therefore, we set to improve the inhibitory potential of the two peptides by implementing a second round of in silico design based on the mono-ORbIT peptide. The peptide opt*-*mono-ORbIT was synthesized to assess its effect on thrombus formation under flow. This peptide showed improved efficacy compared to mono-ORbIT but still did not approach the effects of the blocking CLB-RAg35 mAb. The difference between the peptides and antibody is most likely due to the much higher nanomolar range of affinity of an antibody in comparison to the micromolar range of affinity of the smaller-designed peptides. Moreover, we hypothesized that the reversible peptide-binding will also be more effective in inhibiting the more continued VWF-GPIbα interaction of already-adhered platelets, as we observed with the higher inhibitory effect on aggregation parameters by these peptides than the sub-second interaction of GPIbα of high-shear flowed platelets.

An interesting question is whether the bi-ORbIT peptide has botrocetin-like activity. However, our flow cytometric evaluation indicated that the bi-ORbIT peptide reduced both the botrocetin and ristocetin-induced VWF-binding to platelets. Moreover, we did not see an increase in the basal sample incubated with bi-ORbIT only. We also expect no effect of this peptide on the di or multimeric VWF–VWF interactions because it targets the A1 domain of VWF, which has not been implicated in the multimer assembly of VWF. Protein–protein interactions have been difficult to target using small molecules. Therefore, we in silico designed and synthesized cyclic peptides, which potentially bind to larger inter-protein surface areas in comparison to small molecules [49]. However, our flow cytometry data indicated that the peptide-induced blockade of the interaction between GPIbα and the VWF A1 is relatively weak, which is explained by the likely structural differences present in silico and in vitro. In other words, a slight difference in the calculated epitopes could affect the actual biological effect of the peptide. Accordingly, there is still room for improvement of the in silico design of peptides to better accommodate the complexities of in vitro and in vivo conditions. The advantage of cyclic over linear peptides is based on the knowledge that linear peptides are structurally more flexible, which allows them to also bind with low affinity to the surface or binding pockets of off-target proteins. Hence, linear peptides often have a high tendency to bind to off-target proteins [49]. On the other hand, the higher three-dimensional rigidity of cyclic peptides increases the specificity of binding to their target, i.e., the VWF A1 domain.

Although a complete blocking of the VWF A1-GPIbα binding effect could be detrimental in vivo, as observed clinically with the nanobody caplacizumab, which causes bleeding-related events [23], the designed peptides tested in this study appear to be far from ready to be used in the clinic and several rounds of optimization are still needed to improve their efficacy. Yet, our results show that the combination of in silico and in vitro testing is a useful strategy. We envision that the design of more peptides will lead to greater inhibitory effects in shear-based thrombus buildup.

In the future, after dealing with the initial hurdles with the peptide design and consequent extensive peptide library testing, a future therapy with peptide inhibitors of the VWF A1-GPIbα interaction may ease the thrombotic complications in iTTP, avoiding adverse effects of other therapies. Peptides with mild inhibitory effects may prevent the side effects observed with the caplacizumab nanobody [50]. In iTTP, ultra-large multimers of VWF enhance the formation of GPIbα VWF A1 bonds in the microcirculation due to the presence of autoantibody-mediated clearance/inhibition of ADAMTS13 [23], which implies that targeting of this binding can give beneficial results. On the other hand, it needs to be stated that the charged small peptides are likely to interact with other blood cells and plasma proteins, and are susceptible to degradation by proteases and to clearance, all of which will influence their availability in plasma. Cyclization of peptides, as presented in this work, is an alternative approach to improve the stability and half-life of peptide drugs in plasma and tissue [29]. Nevertheless, for the present class of GPIbα interference peptides, the in vivo half-life still needs to be determined.

### 3.3. Conclusions

The present combined data provide proof-of-principle evidence that in silico design methods can be used to obtain peptides that are able to interfere in the complex ligand–receptor interaction of VWF and platelet GPIb-V-IX, an interaction that is subjected to shear-dependent molecular configuration changes and is partly reproduced with the antibiotic ristocetin as well as the snake venom botrocetin. Future patient treatment with well-designed peptides is considered to be cost-effective, of high target specificity, and with low toxicity in comparison to antibody-based drugs [51]. The peptides designed in this paper can further be modified to improve their inhibitory potential by optimization with repeated rounds of mutagenesis and selection, followed by shear-dependent whole-blood testing.

## 4. Materials and Methods

An extended version of the Materials section is available in the online Data Supplement.

### 4.1. Materials

Collagen-I (Horm-type) derived from equine tendon was obtained from Nycomed (Hoofddorp, The Netherlands). Human placenta-derived collagen-III (C4407) and bovine serum albumin (BSA) were obtained from Sigma-Aldrich (Zwijndrecht, The Netherlands). Human VWF native protein was purchased from ThermoFisher Scientific (RP-43132, Eindhoven, The Netherlands). Fibrinogen was obtained from Sigma-Aldrich (F-4129-16); DiOC_6_ (3,3′-dihexyloxacarbocyanine iodide) from AnaSpec (AS-84715, Seraing, Belgium); and PPACK (D-phenylalanyl-L-propyl-L-arginine chloromethyl ketone) from Calbiochem (520222, Amsterdam, The Netherlands). For fluorescence staining, we used Alexa Fluor (AF)647-conjugated anti-CD62P mAb (304918, Biolegend, London, UK), FITC-labeled anti-fibrinogen mAb (F0111, Dako, Amstelveen, The Netherlands), and AF568-labeled annexin A5 (A13202, ThermoFisher, Eindhoven, The Netherlands). FITC-conjugated sheep anti-human VWF antibody was from Bio-Rad laboratories (AHP062F, Feldkirchen, Germany). Tirofiban (Aggrastat) was from Iroko Cardio (Philadelphia, PA, USA). Ristocetin was from American Biochemical & Pharmaceuticals (London, UK). Other materials were also from Sigma-Aldrich (Zwijndrecht, The Netherlands).

### 4.2. Molecular Dynamics and Binding Free-Energy Calculations

The GPIbα-VWF A1 domain complex and the GPIbα-VWF A1-botrocetin complex (PDB code: 1U0N) were subjected to molecular dynamics (MD) simulations in order to identify key interacting residues at the interface according to a previously established method [37,52]. Herein, AMBER14SB force fields were assigned for proteins and a TIP3P water model was used and added to a 10 Å radius of the molecular surface of the complex. Counter ions (Na^+^ or Cl^-^) were added by replacement of water to neutralize the system. Prior to running MD simulations, the complexes were relaxed by performing two consecutive steps: (i) 4000 steps of energy minimization (2000 steps of the steepest descent, followed by 2000 steps of the conjugate gradient algorithm) to minimize and adjust the positions of water molecules, while partially fixing the positions of proteins by use of a weak force constraint (10 kcal/mol•A^2^), followed by (ii) energy minimization (5000 steps of the steepest descent, followed by 5000 steps of the conjugate gradient algorithm) of the whole complex without applying force constraint to fix the positions of atoms. Initial MD simulation was performed for 100 ps while applying a weak force constraint (10 kcal/mol) to restrain the structure and slowly heat up the system from 0 to 300 K. Subsequently, a free MD simulation phase was performed for 100 ns by setting the temperature at 300 K, the pressure at 1 bar, and the time step at 2 fs with a SHAKE constraint.

The identified key interacting residues of GPIbα with the VWF A1 domain [32] were used as a template to design a range of mono-cyclic peptides. Similarly, two sets of GPIbα and botrocetin residues were connected via different linkers—the disulfide bond or chemical linkage of peptides onto scaffolds [38]—to create bi-cyclic peptides. Several peptides were in silico designed and consequently subjected to MD simulations (5 ns) and binding free-energy (BFE) calculation to predict the relative binding affinity of the designed peptides with the VWF A1 domain. The peptides with the lowest BFE (predicted to be potent inhibitors) were selected for further synthesis and functional characterization.

The BFE of GPIbα-VWF A1, GPIbα-VWF A1-botrocetin, and VWF A1-peptide complexes was calculated by application of the method of molecular mechanics/generalized Born surface area (MM/GBSA). A generalized Born model 8 was applied using default parameter settings. Regarding the GPIbα-VWF A1 and GPIbα-VWF A1-botrocetin complexes, 100 snap shots were extracted from the last 10 ns (90–100 ns) of the molecular dynamics and taken for the BFE calculations. As described before [53], such a molecular dynamics simulation is sufficient to optimize the design of peptides and to estimate the relative BFE. Snapshots of 0–5 ns (500 snapshots) of the in silico VWF A1-peptide complexes were utilized for BFE assessments using the program AMBER16.

### 4.3. Blood and Platelet Isolation

Blood from healthy volunteers was drawn through venipuncture. All volunteers did not receive antiplatelet medication for at least two weeks. All study subjects gave their full informed consent according to the Declaration of Helsinki and the studies were approved by the local Medical Ethics Committee (Maastricht University Medical Centre^+^, NL31480.068.10). Blood was collected into 3.2% trisodium citrate (Vacuette tubes, Greiner Bio-One, Alphen a/d Rijn, the Netherlands). All subjects had platelet counts within the reference range as measured by the Sysmex XN-9000 analyzer (Sysmex, Cho-ku, Kobe, Japan). Platelet-rich plasma (PRP) was isolated from citrate-anticoagulated blood samples, as previously described [10].

### 4.4. Flow Cytometric Assessment of Platelet GPIbα-VWF A1 Interaction

The PRP was diluted at 1:8 with phosphate-buffered saline (PBS) and treated with tirofiban (1.25 µg/mL) to prevent the integrin αIIbβ3-mediated aggregation of platelets. The GPIbα-derived peptides (200 µg/mL) or CLB-RAg35 mAb (5 µg/mL) were pre-incubated with the diluted PRP for 10 min at room temperature. The mixtures were subsequently activated with either ristocetin (0.5 mg/mL) or botrocetin (5 µg/mL) for 6 min at room temperature. The samples were then fixed with 0.5% formaldehyde for 30 min at room temperature. Fixed platelets were washed with 500 µL PBS and pelleted at 800 g for 10 min. Supernatants were then discarded, leaving a residual volume of 100 µL of the platelet-containing pellet. The platelets were then labeled with FITC anti-VWF antibody (1:2000) for 30 min and analyzed using a FACS Canto II (BD Biosciences, San Jose, CA, USA) [10].

### 4.5. Assessment of Shear-Dependent Whole-Blood Thrombus Formation

Microspots of collagen-I, III, and VWF were applied on glass coverslips as 0.5 µL microspots, as described previously [54]. Briefly, three consecutive microspots of collagen -I (100 μg/mL), collagen-III (100 μg/mL), and VWF/fibrinogen (50/250 µg/mL) were applied on washed coverslips. The coated amount was chosen to obtain the maximal platelet adhesion in flow assays [40]. The most active microspot, in this case collagen-I, was located downstream to prevent the cross-activation of platelets during perfusion [40]. Coated coverslips were maintained in a humid chamber for 1 h at room temperature and then blocked with blocking buffer (Hepes buffer pH 7.45, 1% BSA) for 30 min prior to assembly into the Maastricht microfluidic chamber [55].

Samples of 600 μL of citrated whole-blood were pre-incubated for 10 min with either saline, inhibitory CLB-RAg35 mAb (10 μg/mL), or one of the GPIbα-derived peptides (20 μg/mL). The 10 min pre-incubation time was employed to allow homogeneous dispersion of the bicyclic peptide that tended to form aggregates in stock solution. After incubation, 40 μM of PPACK was added and the blood was recalcified (final concentrations: 3.75 mM MgCl_2_ and 7.5 mM CaCl_2_). Whole-blood perfusion through the microspot-containing flow chamber was performed for 3.5 min at a high wall-shear rate of 1600 s^−1^. Staining was then performed by 2 min perfusion with AF647 anti-CD62P mAb (for P-selectin expression), FITC anti-fibrinogen mAb (for integrin αIIbβ3 activation), and AF568-annexin A5 (for phosphatidylserine exposure) [54]. The residual label was removed by postperfusion at 1000 s^−1^ with Hepes buffer pH 7.45, containing 2 mM of CaCl_2_ and 1 U/mL of heparin. All conditions were measured in duplicates using blood obtained from ≥5 healthy donors.

### 4.6. Real-Time Brightfield and Fluorescence Microscopy

Per microspot, two brightfield images and three fluorescence images (overlays) were taken using an EVOS-FL microscope (Life Technologies, Bleiswijk, The Netherlands) equipped with three LED diode cubes (Cy5 626 nm, GFP 470 nm, and RFP 531 nm); an Olympus UPLSAPO 60×oil immersion objective and a 1360 × 1024-pixel CCD camera [41,54] were used. The images were analyzed with standardized, semi-automated scripts in Fiji (ImageJ), calculating surface area coverage (percentage), platelet deposition (P1), thrombus aggregate (P5), and platelet activation (CD62P expression, fibrinogen-binding, and phosphatidylserine exposure; P6–8), and by visual inspection compared with representative images for the morphology score (P2), thrombus contraction score (P3), and thrombus multilayer score (P4), as described [54].

### 4.7. Statistics and Data Analysis

Statistical analysis was performed in GraphPad Prism 8 (GraphPad Software Inc. La Jolla, CA, USA). Heatmaps were generated in R package version 2.3. In the heatmap representation, mean parameter values were univariate-normalized at a scale of 0–10 [54]. Parameter sets for one donor were obtained by averaging the values of duplicate (or triplicate) flow runs of controls and interventions per microspot. Data normality was verified using a Shapiro–Wilk test. Across donors, mean values of control and antibody/peptide runs were compared per each blood sample using a paired Student’s *t*-test. *p*-values below 0.05 were considered to be significant.

## Figures and Tables

**Table 1:** Amino acid sequences of mono-ORbIT and bi-ORbIT peptides with schematic representation of the peptide cyclizations.

Sequence	Peptide	BFE (kcal/mol)
Monocyclic		
GPIbα	^221^QDNAENVYVWKQGVDVKAMTSNVASV^246^	−35 ± 6
Template peptide	H-QDNAENCYVWKQGVDVKAMTSNCAEE-OH Disulfide bond between Cys7 and Cys23	−62 ± 5
Mono-ORbIT	H-DDNAENCYVWKQGDEVRAMRSNCAEE-OH Disulfide bond between Cys7 and Cys23	−76 ± 6
Bicyclic		
Botrocetin	^2070^DVWNKCRF^2077^ ^2089^DYYLIAEYEC^2098^C^2113^-T^2114^	-
GPIbα	^225^ENVYVWK^231^ ^237^KAMTS^241^	
Template peptide	Ac-CDVWNESAFDYYSIAEYECSTENCYVWEPSDTSC-OH Disulfide bond between Cys1 and Cys19 as well as Cys24 and Cys34	−76 ± 9
Bi-ORbIT	Ac-CDVWNESAFDEYSIAESESSECNEYVDEPSYTSC-OH CLIPS(T3 *) connection between Cys1, Cys22, and Cys34	−81 ± 7

* T3 = 1,3,5-tris(bromoethyl)benzene.

**Table 2:** **Assignment of thrombus parameters obtained from end-stage brightfield and fluorescence microscopic images.** Indicated are ranges with mean values per parameter for collagen-I microspots and scaling used for heatmap presentation. Abbreviations: SAC: surface area coverage and PS: phosphatidylserine.

Parameter	Range	Scaling
Brightfield images (platelet adhesion)		
P1 platelet deposition (% SAC)	0–66	0–10
P2 morphology score (0–5)	0–3.8	0–10
Brightfield images (platelet aggregation)		
P3 thrombus contraction score (0–3)	0–2.2	0–10
P4 thrombus multilayer score (0–3)	0–1.9	0–10
P5 thrombus aggregate coverage (% SAC)	0–12	0–10
Fluorescence images (platelet activation)		
P6 CD62P expression (% SAC)	0–50	0–10
P7 fibrinogen-binding (% SAC)	0–21	0–10
P8 PS exposure (% SAC)	0–17	0–10

## Data Availability

All data are included in the manuscript as figures, tables, or supplementary figures.

## Supplementary Materials

The following are available online at https://www.mdpi.com/article/10.3390/ijms23042046/s1.
