# Supplementary material for: Structure-Based Cyclic Glycoprotein Ibα-Derived Peptides Interfering with von Willebrand Factor-Binding, Affecting Platelet Aggregation under Shear
Source: Int J Mol Sci. 2022 Feb 12;23(4):2046. doi: 10.3390/ijms23042046 (PMC8876638; doi:10.3390/ijms23042046)
Supplement: Supplementary file 1 [file ijms-23-02046-s001.zip › Supplemetary for production.pdf]

International Journal of *Molecular Sciences*

MDPI

IJMS

Supplementary Materials and Figures

Structure-based cyclic glycoprotein Ibα-derived peptides interfering with von Willebrand factor binding affecting platelet aggregation under shear

Johana Hrdinova [1,2,+], Delia I. Fernández [1,3,+], Bogac Ercig [1,2], Bibian M.E. Tullemans [1], Dennis P. L. Suylen [1], Stijn M. Agten [1], Kerstin Jurk [4], Tilman M. Hackeng [1], Karen Vanhoorelbeke [5], Jan Voorberg [2], Chris P. M. Reutelingsperger [1], Kanin Wichapong [1,+], Johan W. M. Heemskerk [1,6,+] and Gerry A. F. Nicolaes [1,*]

1 Department of Biochemistry, CARIM, Maastricht University, 6200 MD Maastricht, The Netherlands; [2]Department of Molecular and Cellular Hemostasis, Sanquin-Academic Medical Center, Amsterdam, The Netherlands; [3]Center for Thrombosis and Hemostasis (CTH), University Medical Center of the Johannes Gutenberg University Mainz, 55131 Mainz, Germany; [4]Laboratory for Thrombosis Research, Interdisciplinary Research Facility Life Sciences, Katholieke Universiteit Leuven Campus Kulak Kortrijk, Kortrijk, Belgium. [6]Synapse Research Institute, Kon. Emmaplein 7, 6214 AC Maastricht, The Netherlands.

* Equal contribution

* Correspondence: g.nicolaes@maastrichtuniversity.nl

Supplementary Methods

Solid Phase Synthesis of Mono- and Bi-Cyclic Peptides

The reduced peptides mono-ORbIT (H_2N-DDNAENCYVWKQGDE VRAMR SNCAEE-COOH), *opt*-mono-ORbIT (Ac-EDDNAECAYVEAEGDEARDQRSNCQ DED-COOH), and the linear form of bi-ORbIT (Ac-CDVWNESAFDEYSIAESESS ECNEY-VDEPSYTSC-COOH) were synthesized by manual solid-phase peptide synthesis on a 0.20 mmol scale using the *in situ* neutralization/activation procedure for Boc/Bzl peptide synthesis as previously described,[29] but using HCTU instead of HBTU as a coupling reagent. Glu(OBzl)-PAM resin (0.71 mmol/g), Asp(OBzl)-PAM resin (0.72 mmol/g) and Cys(MeBzl)-PAM resin (0.73 mmol/g) were used as the solid support for mono-ORbIT, *opt*-mono-ORbIT and bi-ORbIT, respectively. After completion of the peptide chain, the resin-bound peptide was washed with dimethylformamide, treated with trifluoroacetic acid (2 × 1 min) to remove the N-terminal Boc group, washed with dimethylformamide, dichloromethane and 1:1 *v/v* dichloromethane/methanol and dried. *Opt*-mono-ORbIT and bi-ORbIT were N-terminally acetylated by treatment with 1:1 acetic anhydride:pyridine for 5 min. The peptides were then sidechain-deprotected and cleaved from resin by treatment with anhydrous HF for 1 h at 0°C, using 4 *vol*% *p*-cresol as a scavenger. Following cleavage, peptides was precipitated in ice-cold diethyl ether. Subsequently, peptides were extracted with 0.1 M sodium acetate buffer (pH 4) containing 6 M guanidine HCl and purified by preparative HPLC (mono-ORbIT and bi-ORbIT. Fractions containing the desired product were identified by UPLC-MS, pooled and lyophilized. *Opt*-mono-ORbIT was extracted from diethyl ether using 50% MeCN in H_2O+0.1% TFA and used in subsequent steps without further purification.

To facilitate formation of the disulfide connection the reduced purified mono-ORbIT was dissolved at 0.3 mg/mL into 1 M guanidine HCl, 0.1 M Tris buffer (pH 8.0), and was stirred for 24 h at 4°C in the presence of 8 mM cysteine and 1 mM cystine as redox couple. Folding progress was monitored by UPLC-MS, appearing as a mass decrease of -2 Da (2 protons, 1 disulfide bound) resulting in a final mass of 3029.32 Da (Supplementary figure 1B), corresponding to the calculated monoisotopic mass of the folded, cyclic mono-ORbIT

peptide. After semi-preparative HPLC, the product-containing fractions were identified by UPLC-MS, pooled and lyophilized.

For the bicyclic peptide, bi-ORbIT, Cys(Mebzl)-PAM resin (0.73 mmol/g) was used as the solid support. Next, the resin-bound peptide was acetylated on N-terminal acetylation using a 1:1 mixture of pyridine/acetic anhydride for 5 min. The peptide was washed with dimethylformamide, dichloromethane and 1:1 *v/v* dichloromethane/methanol and dried. The peptide was further deprotected and cleaved from the resin by treatment with anhydrous HF for 1 h at 0°C, using 4 *vol*% *p*-cresol as a scavenger. Following cleavage, the peptide was precipitated with ice-cold diethyl ether, dissolved in 0.1 M sodium acetate buffer (pH 4) containing 6 M guanidine HCl, and purified by preparative HPLC. Fractions containing the desired product were identified by UPLC-MS, pooled, and lyophilized.

The crude linear peptide of the optimized construct, *opt*-mono-ORbIT, was dissolved at 3 mg/mL into 1 M guanidine HCl, 0.1 M Tris buffer (pH 8.0) and treated with 1% peroxide solution to oxidize the free thiols and form the desired disulfide bridge, Folding progress was monitored by UPLC-MS, appearing as a mass decrease of -2 Da (2 protons, 1 disulfide bound) resulting in a final mass of 3215.10 Da, corresponding to the calculated monoisotopic mass of the folded, cyclic *opt*-mono-ORbIT peptide. After semi-preparative HPLC, the product-containing fractions were identified by UPLC-MS, pooled and lyophilized.

Linear peptide chain of bicyclic peptide, bi-ORbIT, was cyclized by reaction with a 1,3,5-tris(bromomethyl)benzene scaffold (T3) to connect three cysteine residues resulting in a generation of bicyclic peptides as previously described.[40-42] The peptide was dissolved at 2 mg/mL in 50 mM ammonium bicarbonate buffer pH 7.8. One equivalent T3 scaffold (stock solution of 2 mg/mL in acetonitrile) was added to the linear peptide every 30 min until full cyclization was realized. Cyclization progress was monitored by UPLC-MS, resulting in a final mass of 4042.50 Da, corresponding to the calculated monoisotopic mass of the cyclized peptide. After semi-preparative HPLC, product containing fractions were identified by UPLC-MS, pooled and lyophilized.

Semi-Preparative HPLC Purification and Mass Spectrometry

Semi-preparative reversed-phase HPLC was performed by using a Vydac C-18 column (250 x 10 mm, 10 μm). A linear gradient of acetonitrile in water/0.1% trifluoroacetic acid was used to elute the peptide using a flow rate of 12 mL/min. UPLC-MS was performed on a Waters XEVO QTOF G2 mass spectrometer, with an Acquity H-class solvent manager, FTN-sample manager and TUV-detector. The system was equipped with a reversed phase C_{18}-column (Waters, Acquity PST 130 A, 1.7 μm 2.1 x 50 mm), column temperature 40°C. The mobile phases consisted of 0.1% formic acid in water and 90% acetonitrile. FTN-purge solvent was 10% acetonitrile in water. Gradient condition: starting with 10% acetonitrile to 55% acetonitrile in 15 min, absorbance detection at 220 nm wavelength.

Supplemental Figures

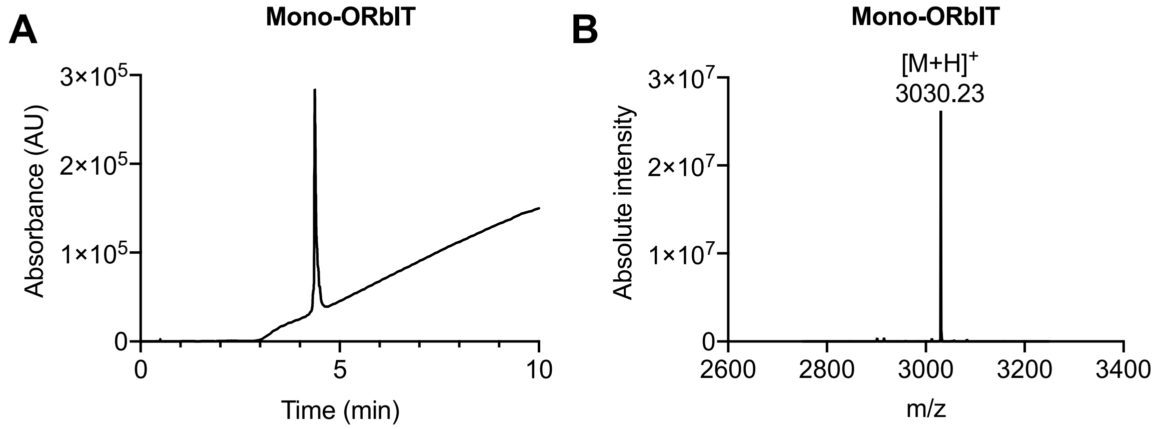

Figure S1. Representative spectra of chemically synthesized and purified mono-ORbIT. A, UV spectrum and B, mass spectrum of mono-ORbIT.

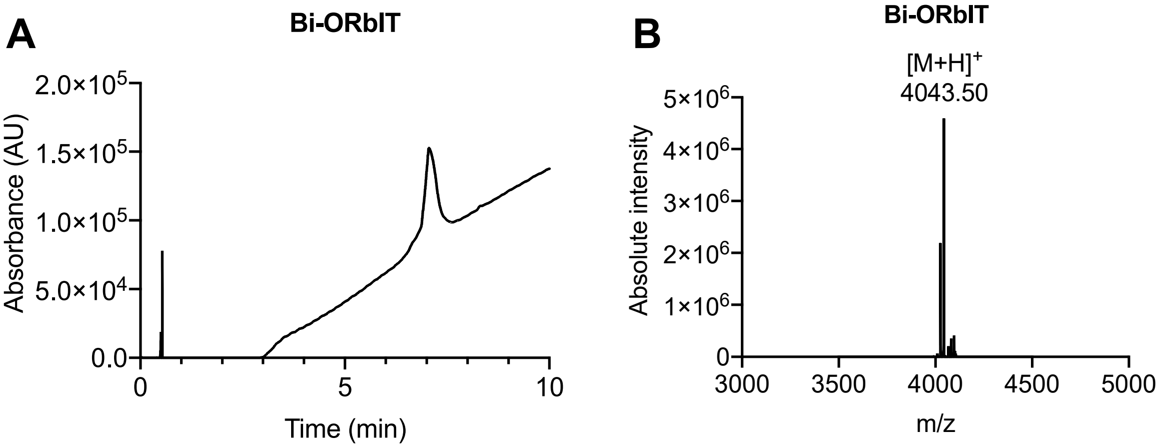

Figure S2. Representative spectra of chemically synthesized and purified bi-ORbIT. A, UV spectrum and B, mass spectrum of bi-ORbIT.

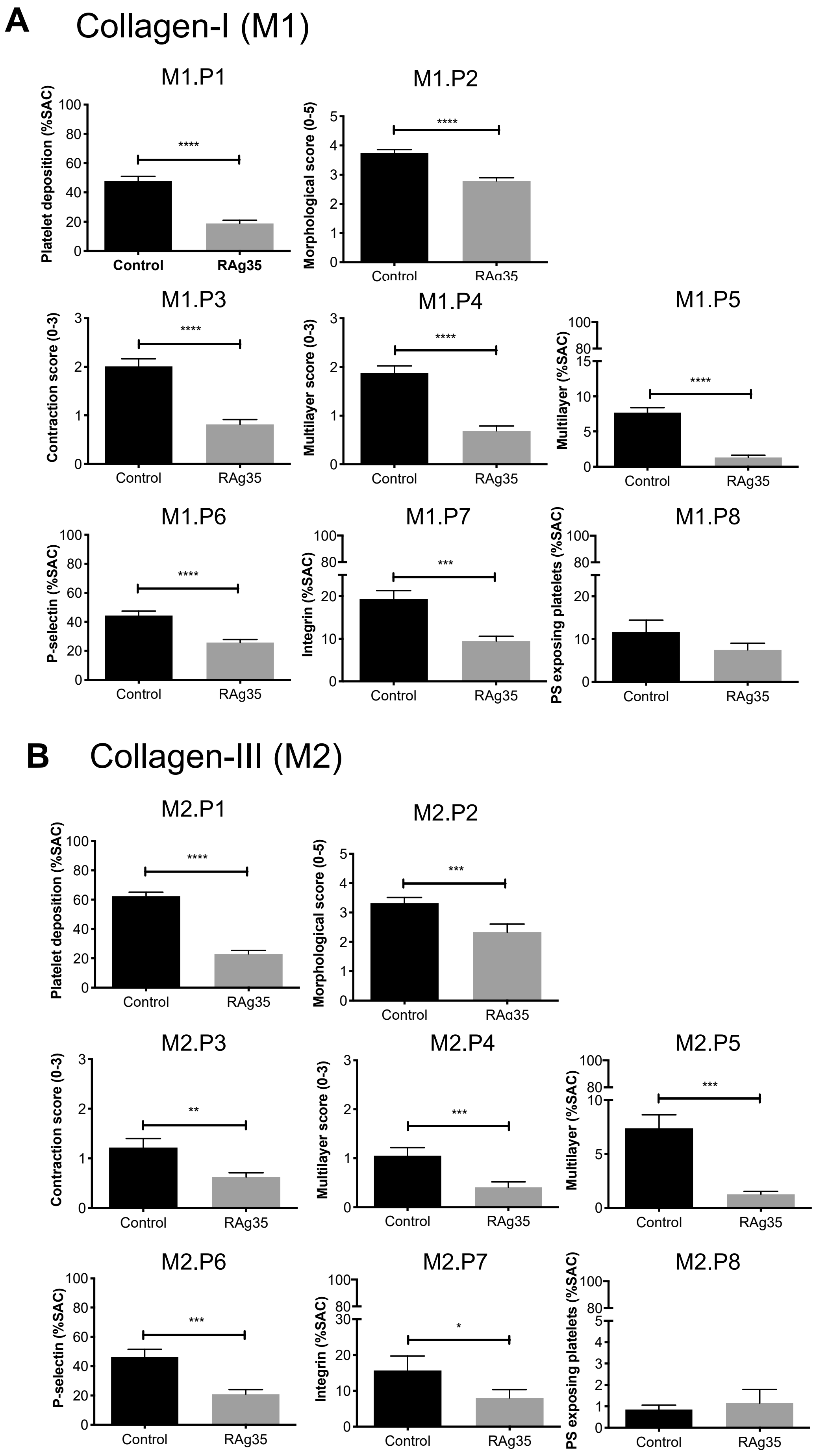
A
Collagen-I (M1)
M1.P1
Platelet deposition (%SAC)
****
Control
RAg35
M1.P2
Morphological score (0-5)
****
M1.P3
Contraction score (0-3)
****
M1.P4
Multilayer score (0-3)
****
M1.P5
Multilayer (%SAC)
****
M1.P6
P-selectin (%SAC)
****
M1.P7
Integrin (%SAC)
***
M1.P8
PS exposing platelets (%SAC)
B
Collagen-III (M2)
M2.P1
Platelet deposition (%SAC)
****
M2.P2
Morphological score (0-5)
***
M2.P3
Contraction score (0-3)
**
M2.P4
Multilayer score (0-3)
***
M2.P5
Multilayer (%SAC)
***
M2.P6
P-selectin (%SAC)
***
M2.P7
Integrin (%SAC)
*
M2.P8
PS exposing platelets (%SAC)

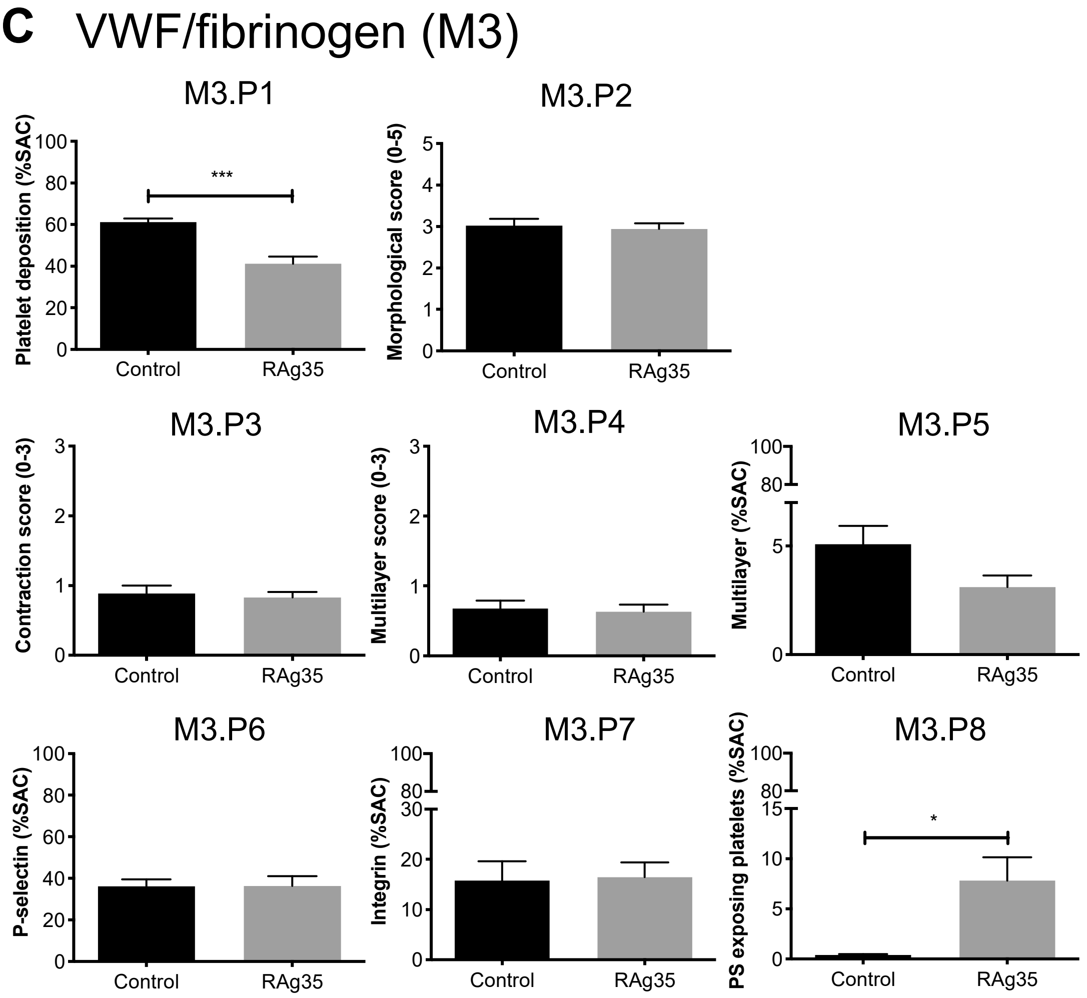

Figure S3. Effect of CLB-RAg35 on thrombus formation at high-shear flow. Microspot thrombus formation was performed, as described in Figure 1. Shown are calculated effects of CLB-RAg35 *vs.* control for microspot M1 (**A**), microspot M2 (**B**) and microspot M3 (**C**), per parameter (P1-8). Paired Student's t-test, *P<0.05, **P<0.01, ***P<0.001, ****P<0.0001 (n=10).

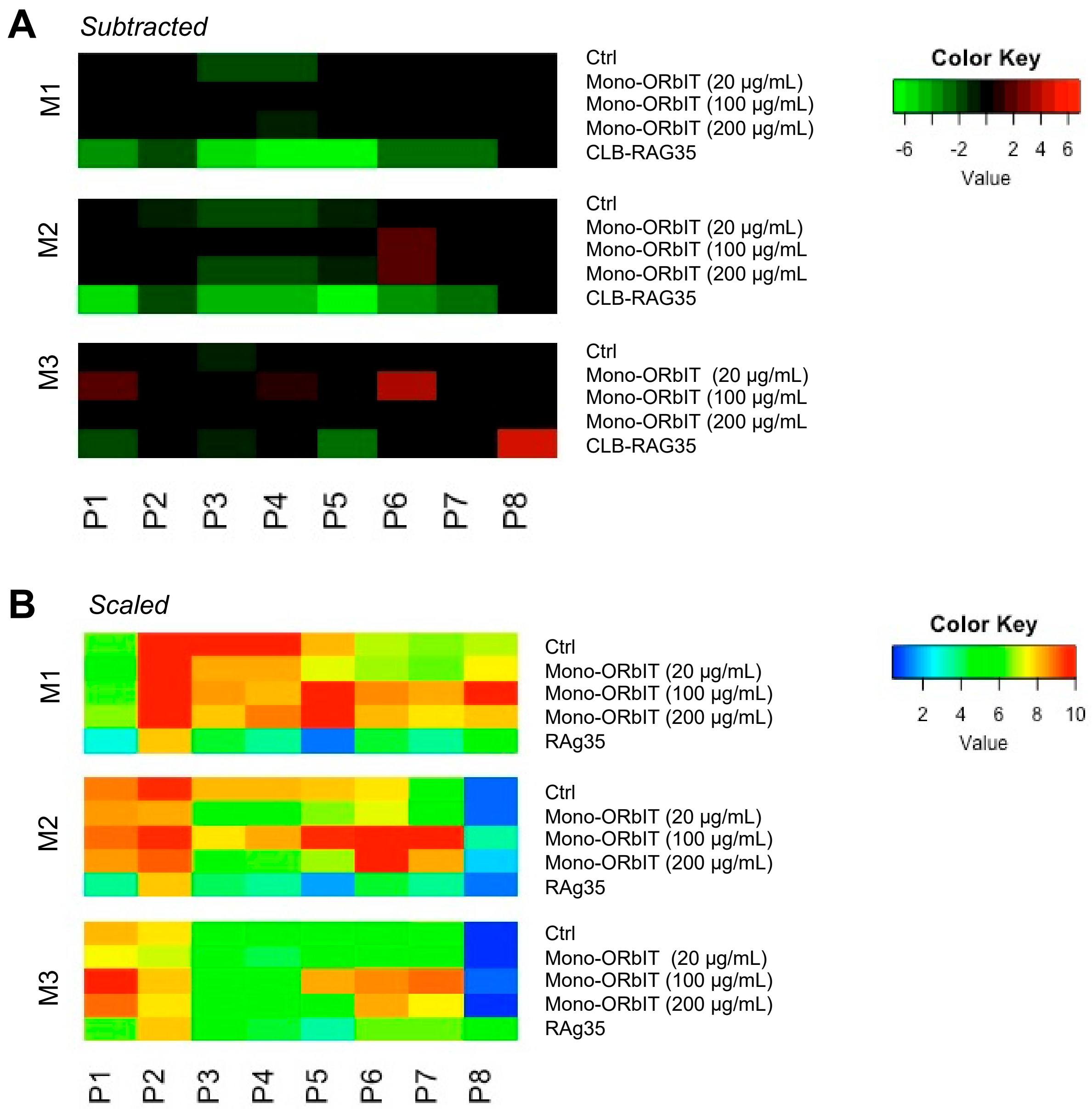

Figure S4. Effects of mono-ORbIT on thrombus formation under flow. Whole blood was preincubated for 10 min in parallel with mono-ORbIT (20, 100 and 200 µg/mL), CLB-RAg35 (Rag35, 10 µg/mL), or control (saline). Samples were perfused over microspots consisting of collagen-I (M1), collagen-III (M2) or VWF/fibrinogen (M3) for 3.5 min at wall-shear rate of 1600 s^{-1}, and then stained, as indicated for Figure 3. Mean values from individual blood samples (*n*=13 donors) were scaled 0-10 per parameter across all microspots M1-3. **A**, Heatmap representation of control-subtracted data. Color coding indicates decrease (green) or increase (red) in comparison to control runs. **B**, Heatmap of mean data per parameter and microspot of thrombus formation. Raw data were scaled on range 0-10 per parameter (P1-8) across all microspots. (n=6-13 donors). See Suppl. Table 3.

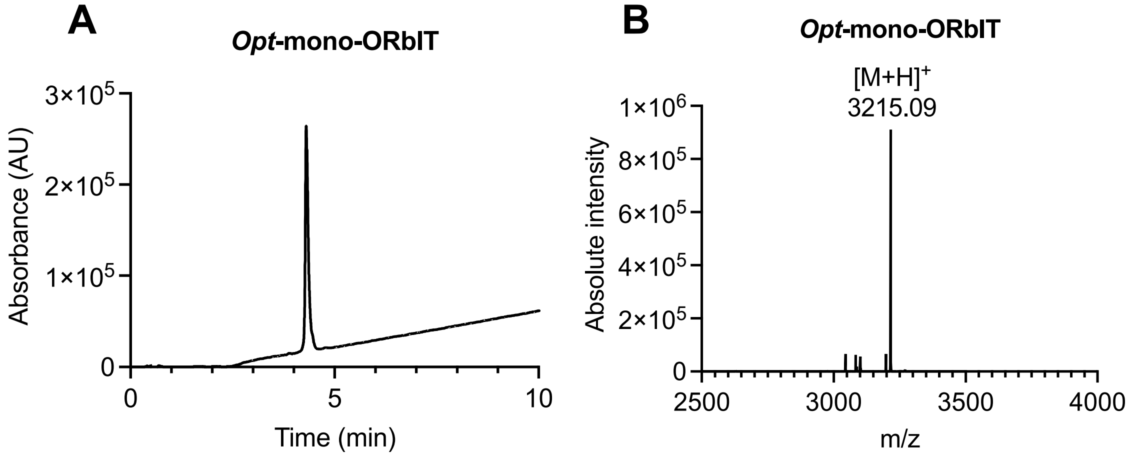

Figure S5. Representative spectra of chemically synthesized and purified *opt*-mono-ORbIT. A, UV spectrum and B, mass spectrum of *opt*-mono-ORbIT.

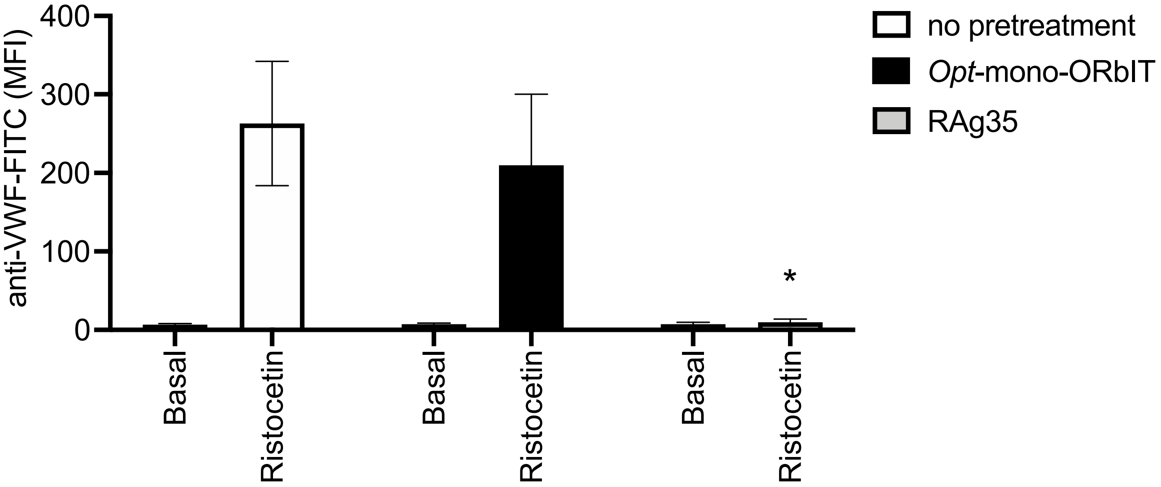

Figure S6. Effect of *opt*-mono-ORbIT on ristocetin-induced platelet binding to VWF. Diluted PRP containing tirofiban (1.25 µg/mL) was pre-incubated with 200 µg/mL *opt*-mono-ORbIT for 10 min at room temperature. Mixture was subsequently activated for 6 min with ristocetin (0.5 mg/mL). Samples were fixed and labeled with FITC-conjugated anti-VWF antibody. Selected gated population of single platelets was used for the analyses. VWF binding calculated as mean fluorescence intensity (MFI). Paired Student's *t*-test, **P*<0.05 (n=3 donors).

Supplemental Tables

Table S1. Raw data per microspot and parameter of control/mono-ORbIT peptide on whole-blood thrombus formation. Data are mean ± SD, *n*=7 donors for mono-ORbIT. For explanation, see Figure 4.

	Condition	P1	P2	P3	P4	P5	P6	P7	P8
M1	Control	44.99±8.62	3.62±0.22	1.75±0.39	1.76±0.30	11.33±4.50	45.22±9.12	22.84±10.73	8.83±4.21
M1	Mono-ORbIT 20 µg/mL	42.37±12.96	3.48±0.37	1.46±0.61	1.46±0.49	10.22±4.77	43.94±12.52	22.12±11.73	9.63±3.58
M1	Mono-ORbIT 100 µg/mL	44.87±6.15	3.58±0.30	1.48±0.32	1.44±0.27	13.47±1.99	55.25±8.94	28.50±6.12	12.69±5.55
M1	Mono-ORbIT 200 µg/mL	46.89±8.76	3.48±0.20	1.40±0.28	1.54±0.25	14.00±1.96	52.99±9.89	26.11±9.92	10.12±3.88
M2	Control	62.16±10.54	3.44±0.53	1.42±0.52	1.44±0.39	11.26±3.60	50.09±9.73	21.05±12.44	1.44±1.12

M2	Mono-OR-bIT 20 μg/mL	59.31±10.19	3.02±0.83	1.04±0.67	1.04±0.65	9.44±4.50	47.14±17.09	19.47±11.29	1.30±0.97
M2	Mono-OR-bIT 100 μg/mL	62.81±7.66	3.42±0.52	1.35±0.50	1.46±0.46	13.31±2.10	64.46±6.06	34.03±10.58	4.44±5.95
M2	Mono-OR-bIT 200 μg/mL	59.85±10.60	3.29±0.37	1.08±0.13	1.13±0.14	9.65±1.68	62.40±3.14	28.34±11.00	2.61±2.48
M3	Control	57.26±17.33	2.79±1.14	0.99±0.54	0.92±0.50	8.17±5.53	35.16±17.35	20.55±14.39	0.54±0.55
M3	Mono-OR-bIT 20 μg/mL	52.39±22.43	2.58±1.00	0.79±0.54	0.75±0.55	7.26±5.36	38.69±21.60	19.78±14.55	0.56±0.59
M3	Mono-OR-bIT 100 μg/mL	70.55±10.30	2.88±0.38	0.98±0.34	1.02±0.32	11.59±3.34	55.76±11.77	30.07±10.71	1.32±1.25
M3	Mono-OR-bIT 200 μg/mL	63.13±8.93	2.79±0.51	0.88±0.31	0.85±0.28	8.42±3.29	53.74±11.86	25.92±14.47	0.68±0.61

Table S2. Raw data per microspot and parameter of bi-ORbIT peptide on whole-blood thrombus formation. Data are mean.

	Condition	P1	P2	P3	P4	P5	P6	P7	P8
M1	Control	46.80±10.18	3.78±0.48	2.19±0.33	2.01±0.7	8.57±2.67	49.75±7.12	20.31±6.55	13.83±2.45
M1	Bi-ORbIT	40.40±8.87	3.45±0.45	1.75±0.5	1.38±0.47	7.89±2.47	50.09±7.12	17.22±8.66	14.14±4.63
M2	Control	58.43±5.33	3.12±0.63	0.95±0.37	0.65±0.22	4.59±2.15	49.55±12.04	15.00±13.89	0.80±0.62
M2	Bi-ORbIT	56.87±6.11	3.03±0.61	0.69±0.19	0.51±0.24	5.16±0.84	50.39±10.94	13.80±10.94	1.69±4.22
M3	Control	51.68±12.37	3.03±0.68	0.72±0.14	0.43±0.19	3.84±1.79	41.14±11.56	16.51±15.22	0.44±0.28
M3	Bi-ORbIT	50.53±10.22	2.80±0.56	0.51±0.11	0.33±0.19	3.58±1.53	14.31±10.38	39.21±14.14	0.44±0.53

± SD, *n*=5 donors bi-ORbIT. For explanation, see Figure 5.

Table S3. Raw data per microspot and parameter of control/Mono-ORbIT2 peptide on whole-blood thrombus formation.

.	Condition	P1	P2	P3	P4	P5	P6	P7	P8
M1	Control	44.35±11.96	3.93±0.37	2.01±0.31	2.17±0.48	15.47±3.86	9.85±2.23	54.25±6.97	28.06±7.16
M1	*Opt*-mono-ORbIT 20 μg/ml	40.07±12.57	3.27±0.28	1.39±0.48	1.45±0.48	11.10±3.63	9.84±4.07	49.91±7.19	24.22±8.43
M1	*Opt*-mono-ORbIT 100 μg/ml	44.99±12.37	3.46±0.47	1.45±0.44	1.55±0.44	12.49±2.65	10.93±5.02	55.05±6.76	30.60±8.68
M1	*Opt*-mono-ORbIT 200 μg/ml	36.45±15.93	3.38±0.33	1.29±0.51	1.50±0.33	15.26±1.76	9.87±6.14	54.76±8.69	26.74±12.58
M2	Control	58.62±15.08	3.90±0.40	1.85±0.45	1.81±0.43	16.78±6.77	1.75±1.04	60.39±10.05	31.27±7.81
M2	*Opt*-mono-ORbIT 20 μg/ml	54.37±16.61	3.25±0.25	1.29±0.42	1.36±0.38	12.32±3.31	2.28±1.79	56.22±9.58	22.08±10.79
M2	*Opt*-mono-ORbIT 100 μg/ml	57.32±17.83	3.27±0.48	1.14±0.38	1.29±0.46	13.09±4.05	3.21±2.52	57.44±8.77	31.53±10.16
M2	*Opt*-mono-ORbIT 200 μg/ml	46.02±20.93	2.83±0.19	0.96±0.31	1.04±0.19	14.56±4.18	1.62±2.11	57.61±11.13	27.79±15.59
M3	Control	65.43±13.27	3.40±0.26	1.36±0.26	1.31±0.28	14.03±7.06	1.28±1.18	54.45±8.09	32.98±8.80
M3	*Opt*-mono-ORbIT 20 μg/ml	61.54±20.01	3.09±0.76	1.20±0.58	1.18±0.59	10.86±5.60	1.06±1.40	51.02±7.21	25.29±8.37

M3	*Opt*-mono-ORbIT 100 μg/ml	63.94±13.54	2.89±0.57	1.00±0.38	1.04±0.36	10.83±5.10	2.97±4.18	53.73±10.58	33.17±9.12
M3	*Opt*-mono-ORbIT 200 μg/ml	50.29±18.69	2.67±0.63	0.75±0.45	0.75±0.50	13.97±6.46	2.58±3.62	55.54±13.96	29.52±18.4

Data are means ± SD, *n*=3-7 donors for mono-ORbIT2.
